# Trauma surgical simulation: discussing the replacement of live animals used as human patient simulators

**DOI:** 10.1186/s41077-024-00279-2

**Published:** 2024-02-12

**Authors:** Cara Swain, Natalia Stathakarou, Pilar Alzuguren, Vincent Lemarteleur, Ryan Moffatt, Klas Karlgren

**Affiliations:** 1 Department of Learning, Informatics, Management & Ethics (LIME), Karolinska Institutet, Stockholm, Sweden; 2 Academic Department of Military Surgery & Trauma, Royal Centre for Defence Medicine (RCDM), Birmingham, UK; 3 Medical Engineering Laboratory, School of Medicine, Universidad de Navarra, Pamplona, Spain; 4 Unité de Recherche en Biomatériaux Innovants Et Interfaces (URB2i), Healthcare Simulation Department, Université Paris Cité, Paris, France; 5 Northern Ireland Medical & Dental Training Agency, Belfast, Northern Ireland; 6 Department of Research, Education, Development & Innovation, Södersjukhuset, Stockholm, Sweden; 7 Faculty of Health and Social Sciences, Western Norway University of Applied Sciences, Bergen, Norway

**Keywords:** Simulation, Trauma, Surgery, Live animal, Debate, Discussion

## Abstract

**Background:**

Despite advances in simulator technology, live anaesthetised animals continue to be used as human patient simulators for medical professionals to practice techniques in the management of surgical trauma. This article describes the process of convening a working group of individuals with a professional interest in simulation to discuss the use of live animals and consider if and how they can be replaced in the future.

**Main body:**

A working group was formed of voluntary attendees to a workshop held at the SESAM 2023 conference. Iterative discussions reflecting on the topic were used to produce statements summarising the working group’s opinions. The working group determined that live animals are used as human patient simulators due to the presence of accurate and responsive physiology in the presence of bleeding, realistic tissue tactility and an emotional response experienced by the learner due to interaction with the animal. They were unable to reach a consensus on replacement, determining that there is currently no single model which is able to provide all the learning aspects which a live animal model can provide. Several suggestions were made regarding development of technologies and pedagogical change.

**Conclusion:**

Replacement of live animals in surgical simulation is not straightforward but should be an aspiration, if possible. For the ongoing development of trauma surgical simulation models, it is important to combine the knowledge, skills and perspectives of medical stakeholders and educators, academic researchers and industry experts in producing alternative options to the use of live animal simulators.

## Background

Simulation is an established adjunct to surgical education experiences gained through clinical practice [1]. There are many different types of simulators available, across the fidelity spectrum, which are used to train varying combinations of technical and non-technical skills [1–3]. Despite advances in simulator technology, live anaesthetised animals continue to be used as human patient simulators for medical professionals to practice surgical techniques [4, 5]; this practice is known as ‘live tissue training’ [4].

The 3Rs, or the replacement, reduction and refinement of animal research, originally proposed in 1959 [6], are accepted worldwide as the best approach to ensure the highest standard of ethical consideration is applied when using animals in scientific research. Due to increasing societal concerns and pressure from animal activist organisations [7], animal use in medical education has decreased over recent years [8, 9]. However, many involved in trauma surgical simulation maintain that live animals are not yet replaceable by alternative simulator models. Two systematic reviews [10, 11] have concluded that there is insufficient data when comparing live animal simulation against other simulation models [10] and that the overall quality of the evidence is poor to moderate [11]. The majority of studies have compared technical skills acquisition and maintenance using different modalities, however, surgical management, particularly in the context of trauma, involves concomitant non-technical skills of decision-making and communication, often situated within a stressful environment. There is a colloquial belief that live animal simulation is superior in this regard. Some studies have attempted to test this hypothesis. While quantitative outcomes have proven to be equivocal [12, 13], qualitative data suggests that live animal training is highly valued by learners [12, 14, 15] due to tissue tactility, physiological responsiveness and psychological engagement during the simulation.

There is an ongoing debate associated with the training requirements of trauma surgery practitioners and the terminal use of animals. Regardless of personal ethical views on the subject, it could be argued that the medical educators and the simulation community should be attempting to honour the 3Rs of animal-dependent science. This article describes the process of convening a working group of individuals with a professional interest in simulation to discuss the use of live animals in trauma surgical simulation and consider if, and how, live animals can be replaced in the future.

## Setting

SESAM, the Society for Simulation in Europe, organises an annual conference. In 2023, this was held from 14–16th June in Lisbon, Portugal, bringing together delegates from a variety of backgrounds across the domains of healthcare, academia and industry.

A workshop entitled ‘Exploring the use of ‘live tissue training’ in learning to manage surgical trauma’ was developed and advertised by authors CS, NS and KK. Proposed details of this workshop were submitted to the SESAM conference committee in advance in the form of an abstract, which underwent peer review. The aims of the workshop were to understand the evidential background in relation to live tissue training; to discuss the requirements for surgical trauma simulation; and to encourage scholarly conversation regarding the use of animals and alternative technologies in surgical simulation. There was a stated intention to use data generated during the workshop to produce a publication for a scientific journal.

## Workshop activity

A working group was formed of voluntary attendees to the advertised workshop. All participants of the workshop were given written information and asked to provide consent for their involvement. The demographics and experiences of the participants were collated anonymously using an online software program. No sensitive information, as described by General Data Protection Regulation (GDPR), was collected.

The workshop began with an introductory seminar delivered by CS and KK regarding surgical simulation. The different modalities which can be used in surgical simulation were briefly discussed, including typical usage and limitations of each. The concept of live tissue training was introduced alongside an outline of the published evidence regarding the educational use of live animals and a summary of the debate. This included descriptive information extracted from the lead authors’ own qualitative systematic review of the literature reporting the educational use of live tissue training in trauma [4] but did not include details of the analysis to minimise the risk of introducing preconceptions. Similarly, the educational outcomes of simulation using live animals or other simulator modalities were deliberately not reported.

Working group participants were subsequently asked to reflect on three reasons why live animals are used within surgical trauma simulation training and then consider potential solutions to replace or refine animal use. A ‘snowball’ process was then used to obtain a consensus of opinion. Ideas and reflections were documented at each stage of the process. The whole group united to discuss their opinions and determine an overall viewpoint, with CS acting as the facilitator. The workshop was delivered in accordance with the details provided to the SESAM conference committee in advance.

## Reflexivity statement

Authors CS, NS and KK are all part of the same research group at Karolinska Institutet in Sweden. CS is a military surgeon and doctoral researcher exploring the use of live tissue training in acquiring competence to manage surgical trauma. NS is an academic lecturer in Health Informatics and a doctoral researcher interested in the use of technology in trauma simulation; KK is an Associate Professor of medical education, with an interest in simulation and is the PhD supervisor of both CS and NS.

We recognise that the various backgrounds and associated biases of the authors from Karolinska Institutet informed the design and delivery of the workshop. CS, NS and KK do not consider themselves in support of, or against, live tissue training, and made efforts to be neutral in their decisions. The working group participants were responsible for producing the data in the form of group discussions that led to the summary statement(s). KK and NS joined the final stage of discussion in the role of facilitator; CS moderated the final group discussion. All attempted to recognise and limit their interactions to avoid influencing the participants’ discussion and ultimate responses.

All workshop participants were invited to co-author this article alongside the facilitators and contribute to ongoing analytical discussions after the conference had ended. The participants who joined the authorship represent surgical practice with a professional interest in medical education and experience with delivery of simulated learning events for undergraduates and postgraduates; a researcher with significant experience in biomedical animal studies and simulator development; and a materials engineer working on developing pedagogical tools. Final decisions about the content of the article and publication were made by CS and KK.

This research is situated in a critical realist paradigm, combining a realist ontology with a constructivist epistemology. Realist inquiry is concerned with ‘what works for whom, under what circumstances, how and why?’ [16]. This working group methodology uses ‘group reasoning’ to explore the features of live animals as human patient simulators and how they influence educational experience to inform ideas and theories about replacement.

## The working group

**Fig. 1:**
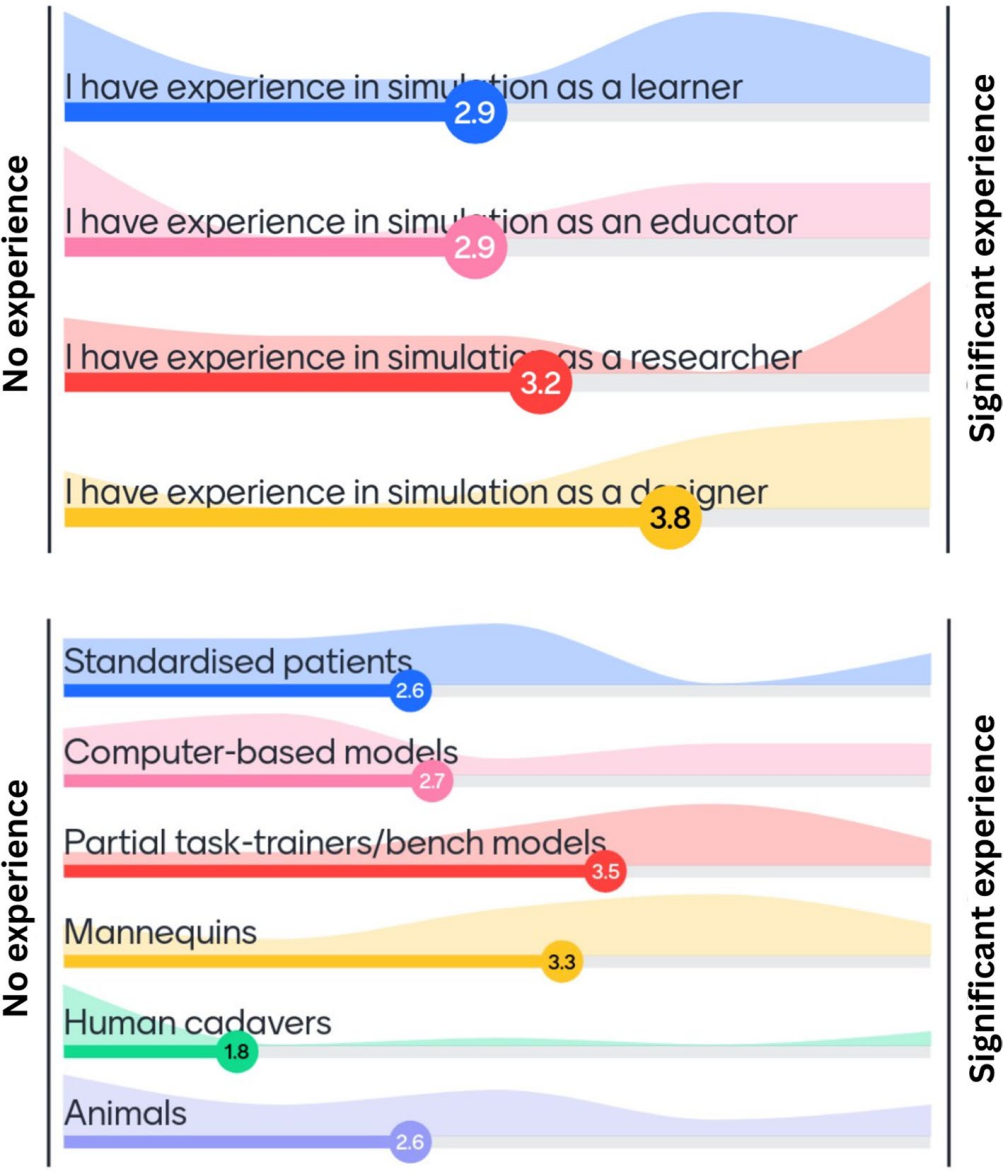
Experience with simulation rated from 1 to 5 by working group participants

The working group consisted of nine workshop attendees and three facilitators from nine different countries. Six worked primarily within the medical field (including surgery, anaesthesia and emergency/pre-hospital trauma) three in academia and three in industry or other fields. The working group was asked to rate their simulation experience in different roles on a Likert scale from 0 to 5; this collective simulation experience can be seen in Fig. 1. The majority of the working group reported higher levels of experience in simulation as a learner and a designer, with partial task trainers and mannequins most commonly used. Two-thirds of the working group had participated or observed in live tissue training previously.

## Summary statements

The working group was asked to reflect on three reasons why live animals are used within surgical trauma simulation training and then consider three potential solutions to replace or refine animal use. The following statements have been synthesised from the workshop discussions and documentation.

### Use of live animals in surgical trauma simulation

*Live animals are used as human patient simulators due to the presence of accurate and responsive physiology in the presence of bleeding, realistic tissue tactility and an emotional response experienced by the learner due to interaction with the animal.*

### Potential solutions to replace or refine animal use

*There is currently no single model which is able to provide all the learning aspects which a live animal model can provide.*

The process of development of the first summary statement regarding the use of live animals in surgical trauma simulation can be seen in Table 1. Data populating this table has been taken from handwritten documentation made during the working group; it is not a complete representation of discussions nor should data be considered as quotes from individual participants.

**Table 1:** Demonstrating the ideas collated and synthesised to form a summary statement describing why live animals are used in trauma surgical simulation

**Round 1—individual reflections**								
Feel of tissues Bleeding Warmth O_2_ saturations Emotive Real time change of physiology	Perfect for learners getting technical skills Haptic feel is very important You learn from real anatomy rather than models	Haemorrhage control + ‘live’, perfused, bleeding Surgical dissection skills Anatomical difference Skeletal fixation Difficult to combine these elements with other simulation modality	To enhance realism and prepare for real life situations To add stress factor To mimic physiology as close as possible	Feeling of tissues Realistic bleeding and bleeding control Realistic preparation of tissue strata	Practice with different kinds of tissues	No realistic simulators Difficult to convince surgeons about shifting to other simulators Impossibility of replicating procedures/acquiring skills if animals are not used	Best haptic fidelity and physical fidelity Major problem = students do not want to be trained on animals	Active haemorrhage Full-size of model ‘Liquidity’ Dynamic behaviour
**Round 2—pairs/group of three**								
Similar to human feedback, graded response, more realistic Anatomy vs physiology—more realistic and important		Better simulates physiology Stress factor: more in line with real trauma management Pinnacle of high-fidelity trauma—for both technical and non-technical skills		Tissue realistic Layers Bleeding			Whole thing with bleeding, visual aspect, smell, combined in one simulation	
**Round 3—two groups, each with a facilitator**								
Tissue and physiology fidelity—dissection, tissue handling, bleeding; lends itself to non-technical skills training in real time Stress response/emotional response/satisfaction of training				Replicate visual aspect, tissue properties, smell Emotional part—influenced by bleeding, and the fact the model is alive				

## Discussion

This article reports the perspectives of a diverse working group including educators, innovators and clinician end-users, regarding how to address the 3Rs. The use of live animals in trauma surgical simulation is an under-discussed topic. Surgical simulator technology has focused on the development of models for laparoscopic, endoscopic and robotic procedures, using a combination of instruments and a two-dimensional screen. Open surgical procedures, however, require the surgeon to use their hands to manipulate tissues in a three-dimensional environment [17]. A significant number of trauma surgery courses use live animals and/or cadavers as human patient simulators [18].

The working group quickly reached a consensus about the likely reasons for using live animal simulators, however, deciding how to replace live animal use was much more challenging. Suggestions for replacement included the following: perfused cadaveric tissue (human and animal); perfused ex-vivo organs; partial task trainers; cut suits; synthetic (hydrogel, silicone or gelatine) models, including the use of 3D-bioprinting; virtual reality; and interactive artificial intelligence (AI). It was agreed that there is not currently a single simulator model on the market which is able to provide all aspects which a live animal model can provide. The working group considered that the current solution to replace live animals as simulators is hybrid. It was stated by one participant that the challenge of replacement is actually about ‘creating life’. If simulation is to engage with the richness of the clinical experience, it must somehow address aspects of the richness and complexity of a true clinical experience [1]. Operating on a live animal shares many features of human surgeries, although with the obvious limitation of anatomical differences. Learners can practice every aspect of an operation, including avoidance and management of complications, with real-time feedback from the model [3].

The physiology of a live animal reacts appropriately to a clinical scenario, whereas synthetic models have a programmed response or require faculty input (for instance, to change parameters to decrease blood pressure and increase heart rate in response to bleeding). Group discussion highlighted the importance of trying to reduce the ‘gap’ between an action performed by the learner and the reaction of the model to promote realism and reduce the requirement for faculty members to be involved within the scenario. It was suggested by one participant that there is the possibility of using AI to achieve this in the future. A recent systematic review of virtual reality and haptic interfaces for open trauma surgery concluded that there was inadequate evidence that these technologies can facilitate training. Pre-training in open procedures using virtual reality technologies could maximise subsequent experiences at cadaver- and live animal-based trauma training. The authors stated that as ‘display technology improves and physical anatomical models advance, the use of live tissue training could be greatly reduced’ [19].

Several working group participants considered that surgical simulators should feature anatomically correct tissues and tissue planes that bleed in a realistic fashion when dissected. Ideally, models should demonstrate anatomical variability and/or pathologies [17] rather than be standardised, to more closely reflect real life. Human cadavers are an alternative option, however, embalmed cadavers typically have poor tissue compliance [3], so fresh frozen cadavers are preferable but more challenging to obtain and store. Technology has been developed to allow for perfusion creating a cadaver with pulsatile, dynamic circulation [20], although these models require expertise and time to prepare, with the tissues becoming oedematous after a few hours [18]. When working with cadavers, the associated financial and logistical barriers are similar to those of live animals.

There are a number of synthetic models on the open market (e.g. SynDaver® surgical model [21]) and wearable simulators (e.g. Strategic Operations Inc. Cut Suit [22]) which have limited capacity for simulated haemorrhage, but no dynamic or responsive circulation. Identifying a dissection plane, due to subtle differences in colour or texture of the tissues, and response to tension is incredibly difficult to simulate [23]. Simulated models for abdominal surgery are reported to have user interface problems and models for vascular procedures have limitations in realism compared to cadavers [18]; it could be argued that similar arguments could be made in comparison to live animals.

The working group additionally recognised that there is a lack of sensory tactile and haptic feedback with many models, which is being addressed in laparoscopic simulators, but not currently by open surgical simulators. It was suggested by one participant that tissue engineering, a set of processes aimed at creating biological tissues, through the use of stem cells and biomaterials, could be used to create tissues by, for example, 3D bio-printing [24], which would allow for appropriate tactile fidelity. Compared with rigid plastic materials, 3D-printed elastomeric organs have elasticity, flexibility and resilience which provide a haptic sensation closer to that of real organs [25].

Another potential option is hydrogels, water-soluble polymers used as cell carriers in the manufacturing process, which have internal structures close to those of living tissue [26]. One aspiration could be customising the composition of hydrogels to approximate the tactile, mechanical and functional properties of a physical simulator, with the aim of reproducing the interaction between a tissue and an instrument [27]. Although progress has been made in this area, the 3D bio-printed models lack responsiveness to electrosurgical instruments, used for precise cutting and to control bleeding through coagulation in many surgical procedures [25]. It was therefore recognised that the use of this technology would be a long-term ambition to improve surgical simulators and replace live animals.

Physicians who learn procedural skills develop embodied knowledge through clinical practice and use visual, kinaesthetic and proprioceptive feedback to refine their technical skills [28]. Surgeons have reported developing ‘sensory semiosis’, the ability to make sense of what they are seeing or feeling [23, 28]. Therefore an aspect of simulation should be learning to interpret visual, tactile and haptic cues into the ‘standardised’ anatomy and ‘expected’ physiology of textbooks. One participant suggested that the use of real (perhaps, warmed) blood in simulation could be beneficial. Blood has specific tactile properties: it has a recognisable colour, is viscid (it feels sticky on your hands!), clots in a specific manner and can even have a characteristic sound. The appearance and sensation of blood evokes an emotional response in many health professionals [4, 15], focusing attention and increasing the requirement to act. Bleeding, especially uncontrolled bleeding, is stressful. Management of physiological response to stress is an important aspect of learning [28].

Fidelity refers to how close the simulation is to reality and encompasses different dimensions: physical, conceptual and emotional [29]. These dimensions combine to produce a perception of realism for a learner, which promotes engagement with the simulation. It is acknowledged that fidelity is complex and can be considered ‘a moving target’ [30] with different dimensions more or less relevant for particular tasks or learner cohorts [30, 31]. Emotional engagement with simulation comprises the learners’ attitudes toward learning and evoked emotional reactions experienced through participating in the activity [29]. The physiology of a live animal contributes to a high level of conceptual fidelity and the sensation of living responsive tissue and active bleeding contributes to physical fidelity. Both of these features may influence the emotional connection experienced with the use of a live animal.

This workshop focused on the use of a live animal as a human patient simulator. The model is one aspect of a simulated learning experience alongside context, type of learner, desired learning outcome(s) and pedagogic design. When considering the replacement of live animal simulators, the working group felt that it was important to consider tailoring the type of model used to the desired learning outcome. Multiple models could be used to address different educational outcomes; there are many instances where a live animal model should not be used, and where other models may be more appropriate. If the aim of the simulation is to practice non-technical surgical skills independently of technical skills, this is possible by using, for example, cut suits or simulated patients. Seniority, prior experience and individual self-confidence influence the educational impact of a learning experience [1]. Novice learners training in basic procedural skills may require simpler, less realistic models in comparison to advanced learners [31]. Fidelity is a spectrum, and no single level of realism will meet all educational needs.

## Limitations

The authors recognise that this small working group was formed from a self-selecting group of voluntary attendees and may not be representative of all views of simulation subject matter experts or the wider simulation community. Subsequent iterations of this workshop might provide different views, which could support or alter the summarised opinions generated.

## Conclusion

A working group of professionals with an interest in simulation considers that live animals continue to be used in training to manage surgical trauma due to a combination of physiology, tissue tactility, bleeding and emotional engagement. Simulation technology has focused on minimally invasive surgery rather than open surgery. Replacement of live animals in surgical simulation is not straightforward. There are multiple potential avenues currently in development, but no single model can presently meet the rationale for using a live animal model. Importantly, any replacement options for live animal simulation need to be cost-effective and sustainable. For the ongoing development of trauma surgical simulation models, it is important to combine the opinions of medical stakeholders and educators, academic researchers and industry experts in producing alternative options for live animals.

## Data Availability

None.

## Abbreviations

AI: Artificial intelligence
GDPR: General Data Protection Regulation
SESAM: Society in Europe for Simulation Applied to Medicine, now known as the Society of Simulation in Europe
